# High-Temperature Mechanochemical Synthesis of Nano-ZrO_2_ for Enhanced Densification and Fracture Toughness in B_4_C Ceramics

**DOI:** 10.3390/ma18102332

**Published:** 2025-05-16

**Authors:** Jingming Xu, Jinchao Jia, Binchuan Li, Daxue Fu, Chunxin Wang, Kuiren Liu, Shicheng Wei, Qing Han

**Affiliations:** 1 School of Metallurgy, Northeastern University, Shenyang 110819, China; 2010749@stu.neu.edu.cn (J.X.); 2410676@stu.neu.edu.cn (J.J.); libc@mail.neu.edu.cn (B.L.); fudx@mail.neu.edu.cn (D.F.); 2 Inner Mongolia First Machinery Group Co., Ltd., China National Weapons Industry Corp., Ltd., Baotou 014030, China; 3 School of Metallurgy and Materials Engineering, Liaoning Institute of Science and Technology, Benxi 117004, China; 4 National Key Laboratory for Remanufacturing, Army Academy of Armored Forces, Beijing 100072, China

**Keywords:** high-temperature mechanochemical technology, nano-ZrO_2_, hot press sintering, B_4_C ceramics

## Abstract

In this investigation, a novel process for the synthesis of nano-ZrO_2_ powders based on high-temperature mechanochemical technology (HTMT) in a short process is proposed and HTMT nano-ZrO_2_ enhancement mechanism as an additive on the properties of B_4_C ceramics was systematically investigated. ZrO(OH)_2_ was used as a precursor, and ZrO_2_-B_4_C composites were prepared by optimizing the ball milling temperature and time in combination with the hot-press sintering technique. The results demonstrated that the high-temperature mechanical force causes the transition temperature of ZrO_2_ from monoclinic to tetragonal crystal system to be decreased to 500 °C. The ZrO_2_ treated by high-temperature ball milling at 600 °C/6 h exhibits lower microstress, higher crystallinity, and a particle size of only about 9.12 nm. HTMT nano-ZrO_2_ effectively controls the size of in situ generated ZrB_2_ particles in B_4_C ceramics, reduces interfacial porosity and grain coarsening, and promotes densification of B_4_C ceramics compared to commercially available nano-ZrO_2_. With the addition of 4 wt% HTMT nano-ZrO_2_, the composite showed optimal comprehensive properties: relative density of 99.75% (2.57 g/cm^3^), fracture toughness of 4.74 MPa/m^1/2^, flexural strength of 266.61 MPa, Vickers hardness of 31.14 GPa, and fracture mode with mixed mechanism of through-crystallization and along-crystallization.

## 1. Introduction

ZrO_2_ represents an outstanding inorganic material that has drawn much attention due to its excellent physical and chemical properties [1]. Its hardness is second only to diamond and exhibits extremely high mechanical strength [2]; meanwhile, its low coefficient of thermal expansion allows it to remain dimensionally stable in high-temperature environments and possesses excellent thermal stability [3]. In addition, ZrO_2_ has excellent chemical stability under extreme conditions such as high temperatures, strong acids, and bases. These properties enable it to show promising applications in ceramics [4], catalysts [5], refractories [6], and biomedicine [7]. ZrO_2_ exists as three crystal types: monoclinic, tetragonal, and cubic, which can be transformed into each other under certain conditions [8]. The monoclinic crystal type is stable from room temperature to 1170 °C, the tetragonal crystal type exists from 1170 °C to 2370 °C, and the elevated temperature is transformed into the cubic crystal type. The unique structure of zirconium dioxide with three crystal types lays a solid foundation for its diversified applications.

Currently, the preparation methods of nano-ZrO_2_ powders mainly include solid–liquid, liquid-phase and vapor-phase methods [9]. Among them, the liquid-phase method is the broadest category of preparation techniques, which mainly covers the co-precipitation method [10], sol–gel method [11], microemulsion method [12], and hydrothermal method [13], etc. The liquid-phase method, which has the advantages of clear chemical reaction process, simple operation, and easy scale-up production, has been widely used in industrial production. However, the nano-ZrO_2_ powders prepared by this method generally suffer from broad particle size distribution, complicated preparation process, and high raw material and energy consumption. With the rapid development of high-performance nano-ZrO_2_ downstream products, the demand for raw materials with better performance continues to increase [14]. Therefore, a short process and low-cost preparation process is urgently needed to produce high-purity, narrow particle size distribution, and highly active zirconia nano-powders to meet the growing market demand.

Mechanochemical processing of zirconium-containing compounds, particularly zircon sand (ZrSiO_4_) and zirconium salts, has emerged as an effective pathway for synthesizing materials such as zirconia (ZrO_2_). Previous studies have demonstrated the versatility of this approach in enhancing reactivity and enabling low-temperature conversion. Puclin et al. [15] systematically investigated the mechanochemical activation of zircon with reductants (e.g., Ca, Mg) and hydroxides. Their work revealed that prolonged ball milling induces structural disordering in ZrSiO_4_, significantly reducing its decomposition temperature from >1750 °C to 840–920 °C during subsequent calcination. This mechanochemically activated route successfully yielded crystalline ZrO_2_ and CaZrO_3_ phases, highlighting the critical role of surface amorphization and nanoscale mixing in promoting solid-state exchange reactions. Complementary research by Ding et al. [16] extended this methodology to zirconium salts. By ball milling ZrCl_4_ with CaO, they developed a nanocomposite that transformed into cubic ZrO_2_ nanoparticles (5–10 nm) embedded in a CaCl_2_ matrix after heating at 300–400 °C. These results underscore that mechanochemical pretreatment enables energy-efficient synthesis compared to conventional high-temperature routes. Collectively, these foundational studies establish mechanochemistry as a robust tool for modifying the reactivity of zirconium compounds. However, the synergistic interplay between mechanochemical and thermal effects in zirconium compound reactions remains underexplored, necessitating further investigation to optimize reaction pathways and material properties.

High-temperature mechanochemical technology (HTMT) provides a novel and efficient way to prepare high-performance ZrO_2_ nano-powders on a large scale. The combination of mechanical energy and thermal energy was realized by low-speed ball milling under high temperature conditions. During the ball milling process, the collision between the grinding balls intensifies the extrusion and collision between the powder particles, which triggers the displacement and reorganization of atoms or molecules on the surface of the particles, destroying the original grain boundary structure and then forming new grain boundaries and defects [17]. This process provides important conditions for the preparation of highly defective and active nano-powders and multicomponent composites. Currently, materials with better wave-absorbing properties such as microcrystalline graphite ZnFe_2_O_4_ [18], core-double-shell structure α-Fe(Si)@Fe_3_O_4_@SiO_2_ composites [19], lithium titanate [20], and other materials such as diffusion reinforcement have been successfully prepared based on the high-temperature mechanically forceful chemical method. However, there is still a gap in the research on the preparation of ZrO_2_ nano-powders using high-temperature mechanochemical techniques, which needs to be further explored and developed.

In this study, ZrO(OH)_2_ was employed as the zirconium source to synthesize nano-ZrO_2_ using a self-designed high-temperature ball milling setup. This approach circumvents the conventional two-step process involving separate dehydration and crystallization steps. By leveraging high-temperature mechanochemical forces, the formation of t-ZrO_2_ was directly promoted without relying on traditional size effects for stabilization, thereby achieving one-step preparation of nano-ZrO_2_ powders. The effects of ball milling temperature and time on the crystalline phase, morphology, and particle size of the synthesized nano-ZrO_2_ were systematically investigated. For comparative analysis, both commercial and HTMT nano-ZrO_2_ powders were utilized as raw materials to fabricate B_4_C-ZrO_2_ composites via an in situ hot-pressing method, aiming to elucidate their distinct impacts on the microstructure and mechanical properties of the composites. This study aims to provide scientific insights and technical support for optimizing the solid-phase synthesis of high-performance nano-ZrO_2_ powders via short-flow processes and their functional applications.

## 2. Experiment

### 2.1. Reagents and Instruments

The raw materials used in the experiments included high-purity ZrO(OH)_2_ (99.9% purity) and nano-ZrO_2_ (99.9% purity), supplied by Ningde Sanxiang Advanced Materials Co., Ltd. (Ningde, China). B_4_C (99.9% purity) and SiO_2_ (99.9% purity) were purchased from Sinopharm Chemical Reagent Co., Ltd. (Shanghai, China). The B_4_C raw material contains B_4_C ≥ 99.9% and the residual carbon ≤ 0.1%. Deionized water was used throughout the experiments. The experimental equipment and instruments comprised a laboratory-built high-energy ball mill (custom-designed, as shown in Ref. [15]) and a ZT-60-22Y vacuum hot-pressing sintering furnace (Shanghai Chenhua Electric Furnace Co., Ltd., Shanghai, China).

### 2.2. Sample Preparation

A predetermined amount of ZrO(OH)_2_ was dried in an electric blast drying oven at 100 °C for 6 h and subsequently ground to obtain the precursor for high-energy ball milling. A total of 100 g of pretreated ZrO(OH)_2_ was combined with zirconia grinding balls (diameters: 6, 8, and 12 mm; mass ratio of 1:1:1; total mass of 2 kg) in a 310S stainless steel ball milling jar. The jar was then loaded into a custom-designed high temperature ball mill. The high temperature ball mill used in this study is the same as that used by Liu et al. [17]. The temperature was raised to the target ball milling temperature (400, 500, 600, or 700 °C) at a heating rate of 5 °C min^−1^. Ball milling was conducted at 90 r min^−1^ for predetermined times (4, 5, 6, or 7 h). After completion, the samples were naturally cooled to room temperature to yield nano-ZrO_2_ powders. The normal distribution function in Origin 2020 software was used to statistically analyze the average grain size of HTMT.

For composite fabrication, ZrO_2_ (commercial or HTBM-synthesized), B_4_C, and SiO_2_ powders were mixed according to the mass fractions listed in Table 1. The mixture was ball milled in a 304 stainless steel jar with zirconia balls and ethanol at 280 r·min^−1^ for 3.5 h to obtain a homogeneous ZrO_2_-B_4_C slurry (sample S2 was specifically selected for supplementary analysis to compare the performance of commercial and HTMT-derived ZrO_2_). The slurry was dried at 100 °C for 6 h, and the resulting powder was compacted in a cylindrical graphite mold (diameter: 40 mm) under a forming pressure of 4600 kN. The green body was vacuum sintered in a sealed graphite heating furnace using the following protocol: (1) heating to 1500 °C at 40 °C·min^−1^, holding for 30 min; (2) applying a uniaxial pressure of 36 MPa while heating to 2240 °C at 100 °C·min^−1^, followed by a 1 h isothermal hold. Finally, the load was removed, and the sample was cooled naturally to room temperature.

### 2.3. Sample Characterization

The density and relative density of the materials were measured using an electronic densitometer (ZMD-2, Shanghai Fangrui Instrument Co., Ltd., Shanghai, China) based on Archimedes’ principle. The sintered ceramic samples were sectioned into specific dimensions using a diamond wire saw, followed by grinding and polishing for subsequent testing. The flexural strength and fracture toughness were determined via three-point bending and single-edge notched beam (SENB) methods, respectively, using a universal testing machine (AG-Xplus 100 kN, Shimadzu Corporation, Kyoto, Japan). Specimens with dimensions of 3 mm (height) × 4 mm (width) × 30 mm (length) were tested under a span of 20 mm with a loading rate of 0.5 mm/min applied along the 3 mm direction. For fracture toughness measurements, a notch (width: ~0.2 mm; depth: 1.4–1.6 mm) was introduced at the center of the tensile surface using wire electrical discharge machining prior to testing. The flexural strength test refers to GBT 16534-2009, and the fracture toughness test refers to GBT 23806-2009. Vickers hardness was evaluated with a hardness tester (HVS-50Z, Laizhou Huayin Testing Instrument Co., Ltd., Laizhou, China). The loading load is 1 kgf, and the indenter is kept under load on the surface of the specimen for 15 s. The Vickers hardness test refers to GBT 16534-2009. For all tests, seven replicates were performed, and the average value was calculated after excluding the maximum and minimum measurements. Thermal analysis of the raw material ZrO(OH)_2_ was performed using a synchronous thermal analyzer (STA 8000, PerkinElmer, Waltham, MA, USA) under an air atmosphere. The temperature range was set from 30 °C to 800 °C with a heating rate of 10 °C min^−1^. The particle size and morphology of nano-ZrO_2_ were observed by TEM (JEM-2010, JEOL, Tokyo, Japan), and the particle size distribution was quantified using Nano-measurer 1.2 software. Phase composition analysis of the products was conducted via XRD (D8 Advance, Bruker, Munich, Germany) with Cu-Kα radiation, scanning from 10° to 80° at a rate of 0.02° s^−1^. The average crystallite size (*D*) and microstrain (ε) were calculated using the Debye–Scherrer equations [21]:

(1) $$D\;=\frac{K\lambda }{\beta \cos\theta }$$

(2) $$\varepsilon \;=\frac{\beta }{4\tan\theta }$$

where *K* is the Scherrer constant (0.89), *β* is the full width at half maximum (FWHM) of the diffraction peak, *θ* is the Bragg angle, and *λ* is the X-ray wavelength (0.15406 nm).

The bulk density of sintered ceramics was measured using a gas pycnometer (AccuPyc 1340, Micromeritics, Norcross, GA, USA). The relative density was determined as the ratio of experimental bulk density to the theoretical density calculated via the rule of mixtures based on the final composition. Phase identification was further verified by XRD with Cu-Kα radiation at 40 kV, scanning from 5° to 90° at 10° min^−1^. The microstructure of sample surfaces and fracture cross-sections was examined by SEM and EDS (Sigma 500, ZEISS, Oberkochen, Germany) in secondary electron mode. For grain size analysis, sintered ceramics were polished to a thickness of 0.5 mm using diamond abrasive paper and observed under an optical microscope (MDS400, Olympus, Tokyo, Japan). Before observing with an optical microscope, the boron carbide ceramics were electrolytically etched. The specific parameters are as follows: the electrolyte is a KOH solution with a concentration of 0.1 mol/L; and the boron carbide ceramics serve as the anode, the electrolytic current is 0.1 A, and the electrolysis time is 60 s. The software Nano Measurer was used to mark the grain boundaries of B_4_C in the optical micrographs and calculate its grain size by the method of the diameter of the equivalent circle. Vickers hardness of B_4_C ceramics was measured using a microhardness tester (Tukon 2500, Wilson Hardness, Binghamton, NY, USA) with a 9.8 N load applied for 15 s.

## 3. Results and Discussion

### 3.1. Thermogravimetric-Differential Thermal Analysis of ZrO(OH)_2_

**Figure 1:**
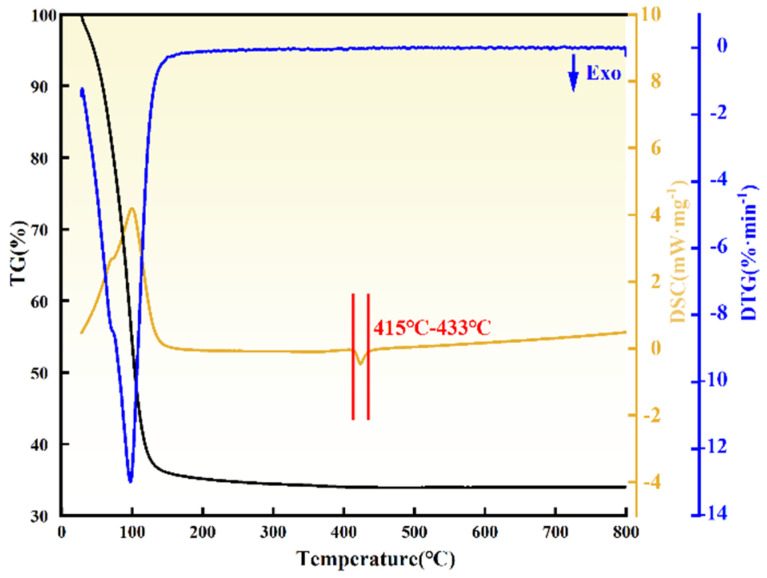
TG-DSC-DTG plot of ZrO(OH)_2_.

The thermogravimetric-differential thermal analysis (TG-DTA) results of ZrO(OH)_2_ are shown in Figure 1. The thermal decomposition process of ZrO(OH)_2_ exhibited a single-stage weight loss behavior. As indicated by the TG curve, significant weight loss (43.3%) occurred between 30 °C and 130 °C, with the maximum weight loss rate observed at ~98 °C. This stage corresponds to the desorption of physically adsorbed water molecules from the surface of ZrO(OH)_2_, driven by overcoming van der Waals forces. From 130 °C to 415 °C, a gradual mass decline was attributed to the removal of internal hydroxyl groups, accompanied by the initial formation of the ZrO_2_ crystalline phase. A distinct exothermic peak appeared between 415 °C and 433 °C, corresponding to the cleavage of Zr–OH bonds and subsequent nucleation of ZrO_2_ crystals. Above 433 °C, the TG curve stabilized, indicating the completion of ZrO(OH)_2_ decomposition and the formation of thermally stable ZrO_2_ [22].

### 3.2. Effect of Ball Milling Temperature

**Figure 2:**
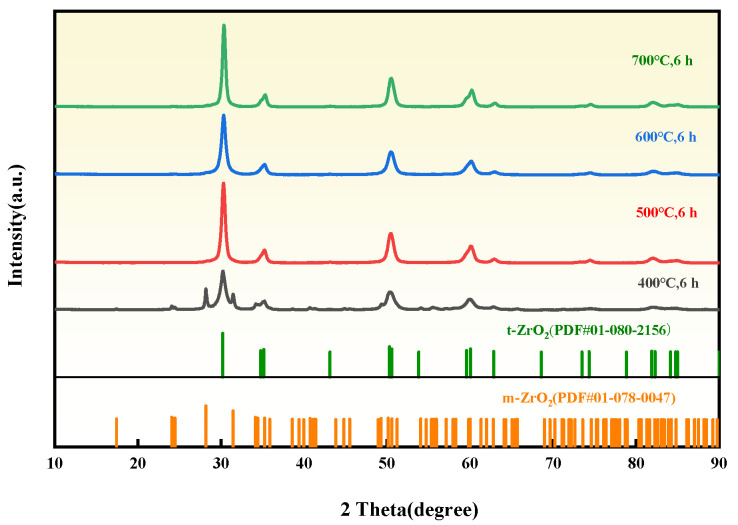
XRD patterns of ZrO_2_ prepared by different ball milling temperatures.

**Figure 3:**
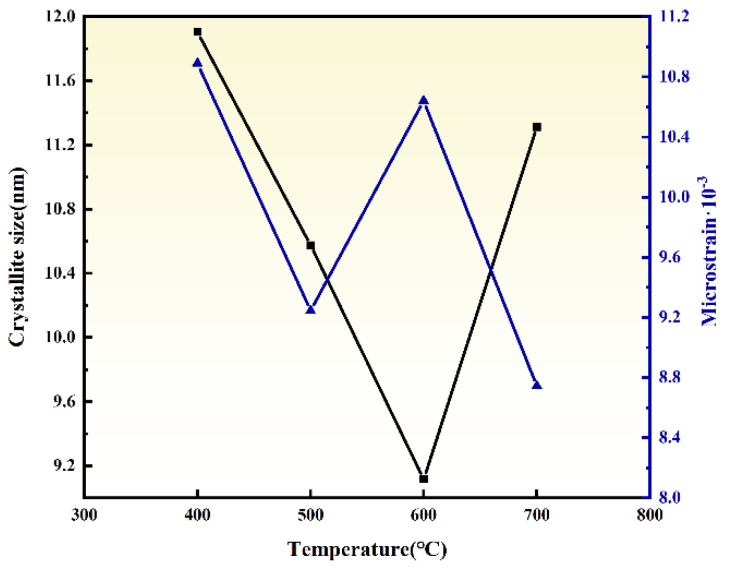
Crystallite size and microstrains plot of ZrO_2_ prepared by different ball milling temperatures.

The influence of ball milling temperature (400, 500, 600, and 700 °C) on the crystallinity, particle size, and microstrain of ZrO_2_ was investigated by ball milling dried ZrO(OH)_2_ for 6 h under each condition. The XRD patterns of ZrO_2_ (Figure 2) demonstrate that the sample ball milled at 400 °C for 6 h contains both m-ZrO_2_ and t-ZrO_2_ phases, with weak diffraction intensities indicative of low crystallinity. As the ball milling temperature increases to 500 °C, the monoclinic phase is transformed into the tetragonal phase, and further temperature elevation (500–700 °C) significantly enhances the crystallinity of t-ZrO_2_. The transition from monoclinic to tetragonal ZrO_2_ under HTMT conditions aligns with prior studies on mechanochemically driven phase stabilization. As demonstrated by Suryanarayana [23] the combination of shear stress and thermal activation reduces the energy barrier for t-ZrO_2_ formation, enabling its retention at room temperature despite subcritical crystallite sizes. The particle sizes and strain calculated via the Debye–Scherrer equations ((h k l) = (1 0 1), (1 1 2), and (2 1 1)) are summarized in Figure 3. Under 6 h of ball milling at 400–700 °C, the ZrO_2_ particle sizes are 11.91, 10.58, 9.12, and 11.31 nm, while the corresponding microstrain are 10.89, 9.25, 10.64, and 8.74 × 10^−3^, respectively. Notably, the particle size exhibits a non-monotonic trend, decreasing initially and then increasing with rising temperature. This behavior is attributed to the ~7% volume contraction during the m-ZrO_2_-to-t-ZrO_2_ phase transition, which generates additional internal stress within particles. At lower temperatures (400–500 °C), sluggish atomic diffusion and limited grain boundary migration allow the dominant impact and shear forces from milling media to refine ZrO_2_ particles effectively. Conversely, at elevated temperatures (600–700 °C), enhanced grain boundary diffusion facilitates particle coalescence, leading to increased particle sizes. Simultaneously, the reduced influence of milling-induced lattice distortion results in diminished microstrain. Considering the interplay between particle refinement and phase stability, a ball milling temperature of 600 °C is identified as the optimal condition.

### 3.3. Effect of Ball Milling Time

**Figure 4:**
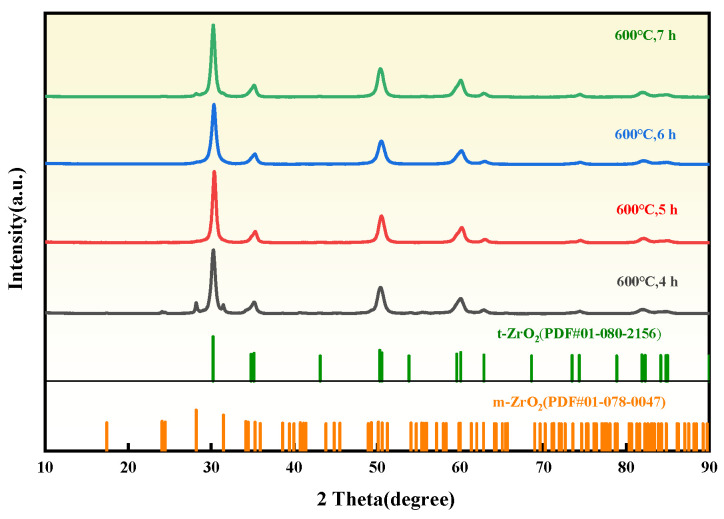
XRD patterns of ZrO_2_ prepared by different ball milling time.

The influence of ball milling time (4, 5, 6, and 7 h) on the phase composition, particle size, and microstrain of ZrO_2_ was investigated by ball milling dried ZrO(OH)_2_ at 600 °C. As shown in the XRD patterns of ZrO_2_ (Figure 4), the sample ball milled for 4 h at 600 °C contains both m-ZrO_2_ and t-ZrO_2_ phases, with weak diffraction peak intensities indicating low crystallinity. Prolonging the milling time to 5 h induces a transition from the monoclinic to the tetragonal phase, and further extension of milling time significantly enhances the crystallinity of t-ZrO_2_. The dominance of t-ZrO_2_ after prolonged milling (6 h) is consistent with the defect-mediated stabilization mechanism proposed by Garvie [24], where lattice strain and dislocation networks suppress the m-ZrO_2_ reconstruction during cooling.

**Figure 5:**
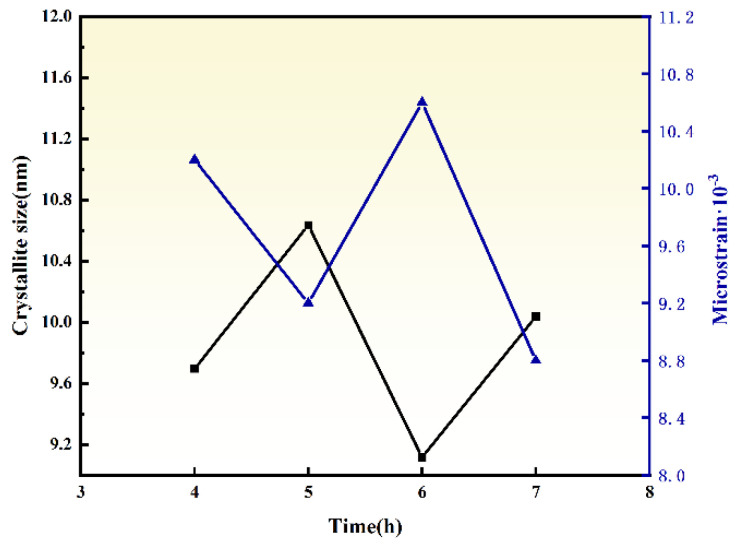
Crystallite size and microstrains diagram of ZrO_2_ prepared by different ball milling time.

The particle sizes and strain calculated via the Debye–Scherrer equations are summarized in Figure 5. Under 600 °C ball milling for 4–7 h, the ZrO_2_ particle sizes are 9.70, 10.62, 9.12, and 10.03 nm, while the corresponding microstrain are 10.2, 9.2, 10.6, and 8.8 × 10^−3^, respectively. Notably, after 6 h of milling, enhanced thermal vibration of Zr atoms facilitates grain boundary migration and rearrangement, leading to increased particle size (9.12 nm) and reduced microstrain (10.64 × 10^−3^). This phenomenon suggests that prolonged thermal–mechanical coupling promotes particle coalescence and stress relaxation. At 600 °C with 6 h of milling, ZrO_2_ exhibits the smallest particle size (9.12 nm) and highest microstrain (10.64 × 10^−3^). Considering the balance between phase stability and particle refinement, a ball milling time of 6 h is identified as optimal.

**Figure 6:**
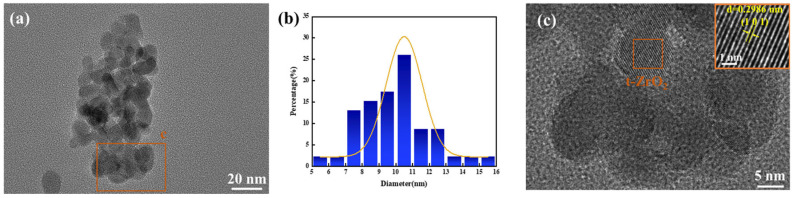
Ball milling at 600 °C for 6 h: (**a**) ZrO_2_ TEM image, (**b**) ZrO_2_ particle size distribution, and (**c**) HRTEM image.

The TEM images, particle size distribution, and HRTEM analysis of the ZrO_2_ powders synthesized under these conditions are presented in Figure 6. As shown in Figure 6a, the ZrO_2_ particles exhibit irregular clusters with good dispersion and uniform spherical morphology. The particle size distribution histogram (Figure 6b) reveals a narrow size distribution centered at ~10 nm. The HRTEM image (Figure 6c) displays distinct lattice fringes, confirming high crystallinity and well-defined grain boundaries. The interplanar spacing measured in the orange-boxed region is 0.2986 nm, corresponding to the (101) plane of t-ZrO_2_, which aligns with the XRD analysis results.

### 3.4. Phase Characterization of ZrO_2_-B_4_C Composites

**Figure 7:**
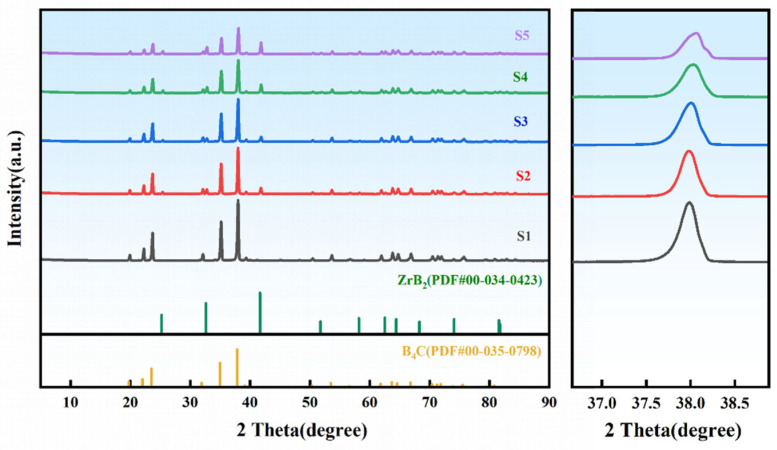
XRD patterns of B_4_C samples with different ZrO_2_ additions.

The XRD patterns of B_4_C ceramics with varying HTMT-ZrO_2_ additions sintered at 2240 °C are shown in Figure 7. The rhombohedral B_4_C phase remains dominant in all samples, with B_4_C (Sample S1) exhibiting characteristic diffraction peaks at 2*θ* = 19.7° (101), 22.0° (003), 23.5° (012), 34.9° (104), and 37.8° (021). Upon introducing 2 wt% commercial nano-ZrO_2_ (Sample S2), weak ZrB_2_ peaks emerge at 2*θ* = 32.6° (100) and 41.6° (101). Above 2000 °C, the following interfacial reaction will occur between ZrO_2_ and B_4_C [25]:

4ZrO_2_(s) + 5B_4_C(s) → 5ZrB_2_(s) + 4CO(g) + 6BO(g) (3)

Notably, the ZrB_2_ peak intensity in Sample S3 (2 wt% HTMT-ZrO_2_) increases by 23% compared to S2 (peak area ratio $I_{{ZrB}_{2}}:\;I_{{ZrB}_{2}}=0.15\;:0.12$), attributed to the enhanced reactivity of HTMT-ZrO_2_ due to optimized surface hydroxylation and reduced agglomeration. Increasing the HTMT-ZrO_2_ content from 2 to 4 wt% (Sample S4) significantly promotes ZrB_2_ formation [26], accompanied by a broadening of the B_4_C (021) peak FWHM from 0.26° to 0.28°, indicative of ZrO_2_-induced grain refinement. Further increasing HTMT-ZrO_2_ to 6 wt% (Sample S5) intensifies ZrB_2_ generation but reduces the B_4_C (021) FWHM to 0.25°, suggesting that ZrB_2_-mediated interfacial slip becomes the dominant stress relaxation mechanism.

### 3.5. Effect of ZrO_2_ Content on the Microstructure of B4C Ceramics

**Figure 8:**
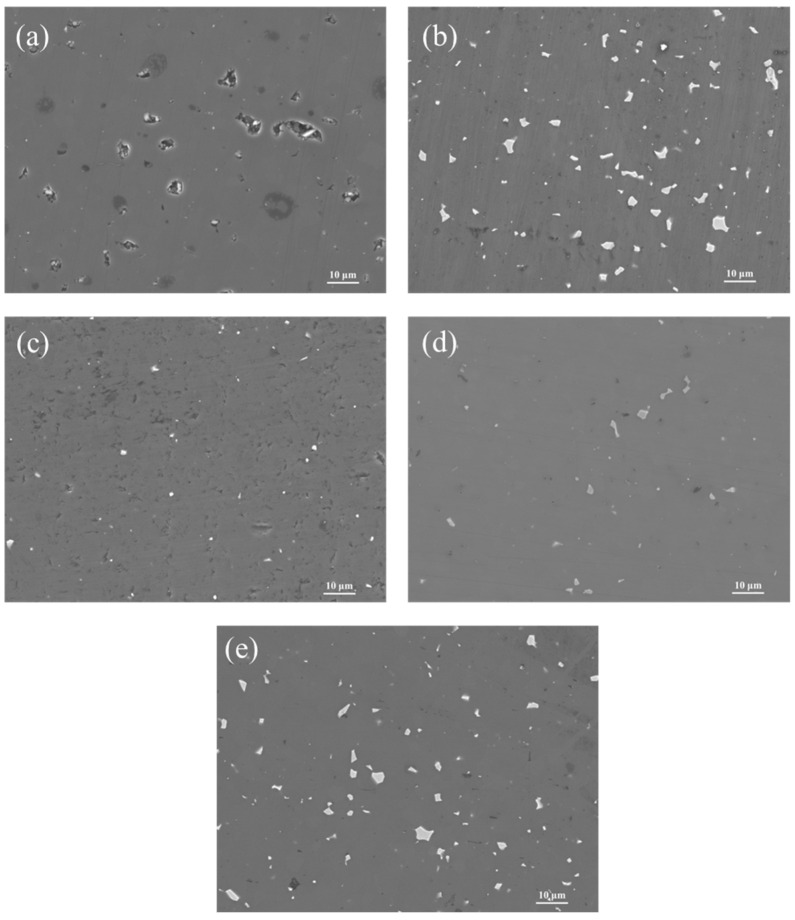
Surface SEM images of B_4_C samples with different ZrO_2_ additions, (**a**–**e**) Samples S1–S5.

The SEM images of B_4_C ceramics with varying ZrO_2_ additions sintered at 2240 °C are presented in Figure 8. As shown in Figure 8a, Sample S1 exhibits a porous structure with irregular pores (1–5 μm) due to incomplete densification caused by insufficient sintering activity. Sample S2 demonstrates improved densification compared to S1 but contains localized irregular platelet-like ZrB_2_ particles and non-uniform particle size distribution, attributed to the poor dispersion of commercial nano-ZrO_2_. In contrast, Sample S3 achieves a homogeneous microstructure with significantly reduced ZrB_2_ agglomerates of smaller dimensions (<500 nm), where the enhanced dispersion of HTMT-ZrO_2_ strengthens interfacial bonding [26]. Sample S4 displays a smooth surface with further reduced porosity, minimal ZrB_2_ agglomeration, and no visible microcracks. However, Sample S5 shows distinct platelet-like ZrB_2_ agglomerates, indicating that excessive ZrO_2_ addition triggers coarsening of the secondary ZrB_2_ phase, thereby degrading mechanical performance.

**Figure 9:**
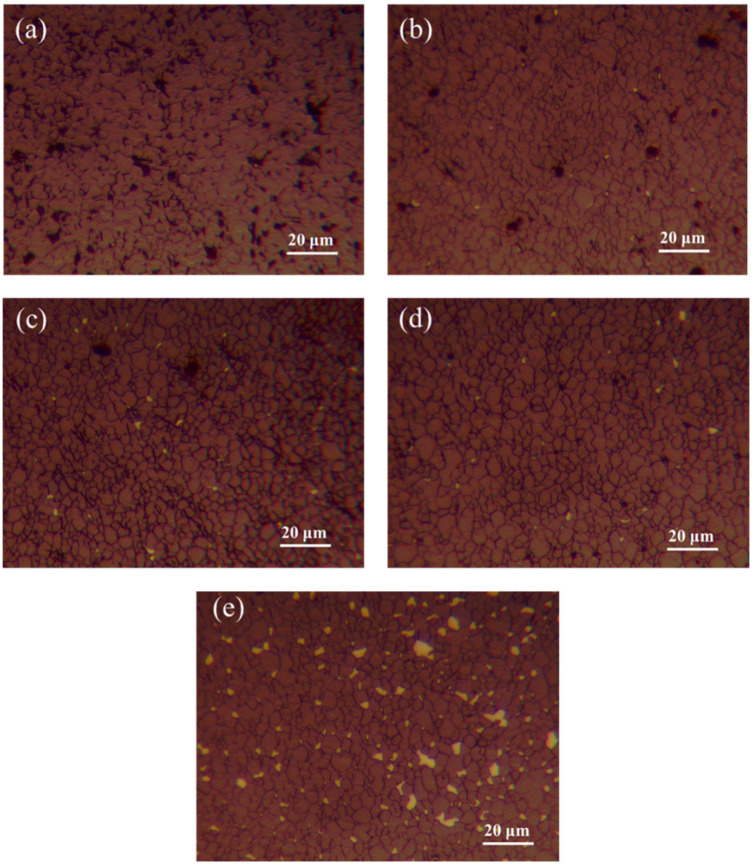
Etched metallographs of B_4_C samples with different ZrO_2_ additions, (**a**–**e**) Samples S1–S5.

The etched metallographic images of B_4_C ceramics with varying ZrO_2_ additions sintered at 2240 °C are shown in Figure 9. As illustrated in Figure 9a, Sample S1 exhibits uniform grain distribution (average size: 9.7 ± 0.5 μm) but contains numerous intergranular pores (diameter: 5–8 μm, porosity: 8%), indicating poor densification. In Sample S2, the porosity decreases to 6.2%, with reduced grain size (7.4 ± 0.5 μm) and localized ZrB_2_ enrichment at B_4_C grain boundaries. In contrast, Sample S3 achieves finer grains (5.5 ± 0.3 μm) and lower porosity (3%), where in situ-formed ZrB_2_ particles at B_4_C grain boundaries inhibit grain boundary migration and mass transfer, thereby refining the grains [27]. Sample S4 demonstrates a reinforcing network of ZrB_2_ platelets, which enhances localized dislocation motion and significantly reduces pore size (diameter: 2–4 μm, porosity: 2.8%) through reactive liquid-phase sintering. For Sample S5, the porosity drops below 1%, with optimized densification due to second-phase strengthening by HTMT-ZrO_2_-derived products; however, localized Zr-rich zones are observed.

**Figure 10:**
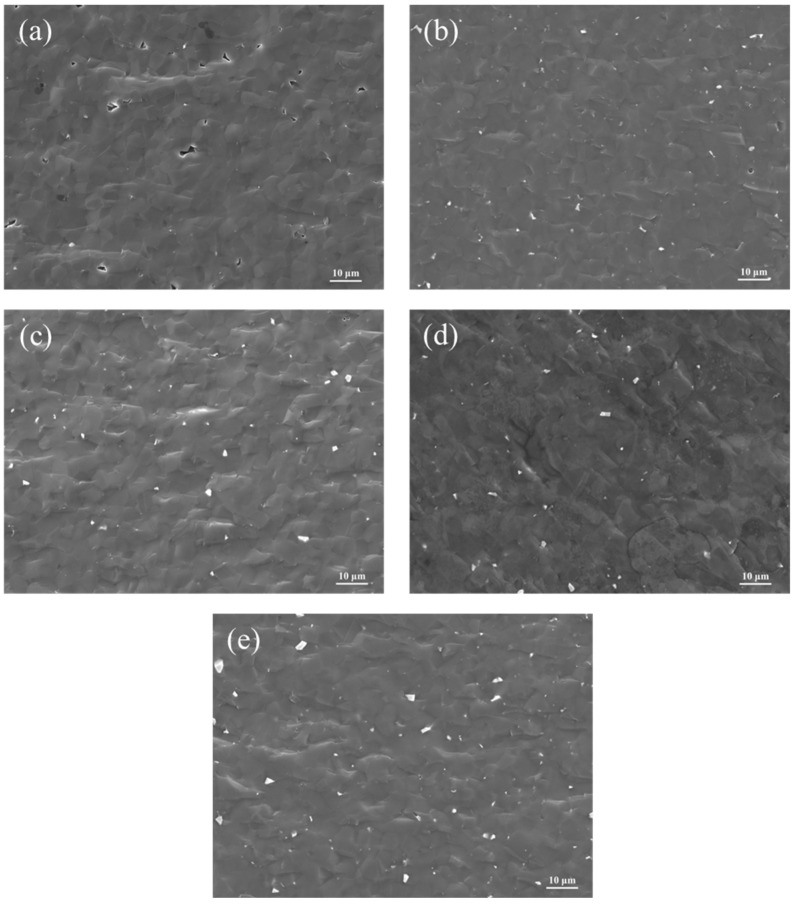
SEM images of fracture surfaces of B_4_C samples with different ZrO_2_ additions, (**a**–**e**) Samples S1–S5.

The fracture surface SEM images of B_4_C ceramics with varying ZrO_2_ additions sintered at 2240 °C are shown in Figure 10. As observed in Figure 10a, Sample S1 displays a relatively smooth fracture surface dominated by transgranular fracture, accompanied by sparse pores due to incomplete densification. In contrast, Samples S2–S5 exhibit a mixed fracture mode of transgranular and intergranular failure with minor porosity. This is attributed to CO gas generation during the in situ reaction between ZrO_2_ and B_4_C to form ZrB_2_. At elevated temperatures, the grain boundary migration rate of B_4_C exceeds the venting rate of CO gas, resulting in trapped pores. As the HTMT-ZrO_2_ content increases, its sintering-aid effect intensifies, leading to smoother fracture surfaces and a higher proportion of intergranular fracture [28], which correlates with enhanced interfacial reactions and improved densification.

### 3.6. Effect of ZrO_2_ Content on the Mechanical Properties of B_4_C Ceramics

**Figure 11:**
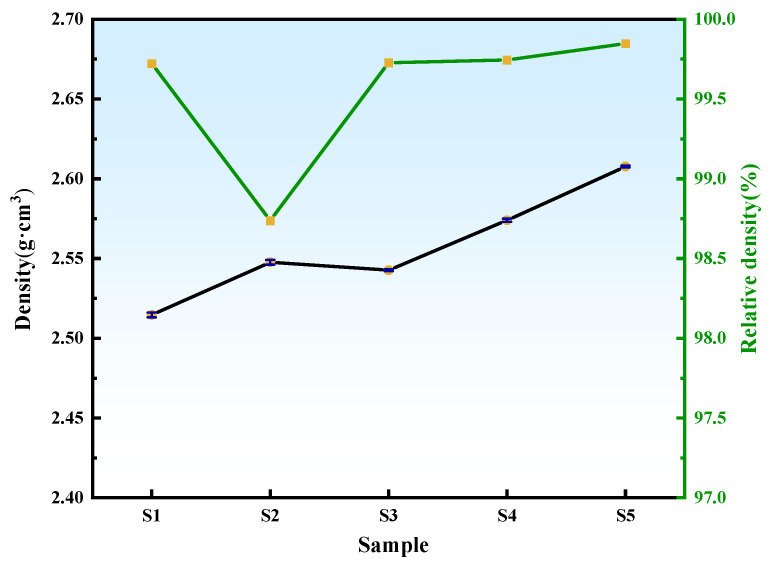
Density and relative density plots of B_4_C samples with different ZrO_2_ additions.

The density and relative density of B_4_C ceramics with varying ZrO_2_ additions sintered at 2240 °C are shown in Figure 11. Sample S1 exhibits a density of 2.51 g·cm^−3^ and a relative density of 99.72%, confirming the efficacy of SiO_2_-assisted liquid-phase sintering. For Sample S2, the density increases to 2.54 g·cm^−3^; however, agglomeration of commercial nano-ZrO_2_ particles generates large trapped pores, slightly reducing the relative density to 98.73% [29]. With increasing HTMT-ZrO_2_ content, the density of B_4_C ceramics progressively rises. The superior dispersion of HTMT-ZrO_2_ minimizes interfacial porosity caused by agglomeration, while the in situ formation of uniformly distributed ZrB_2_ platelets synergizes with the SiO_2_ liquid-phase sintering effect to enhance relative density.

**Figure 12:**
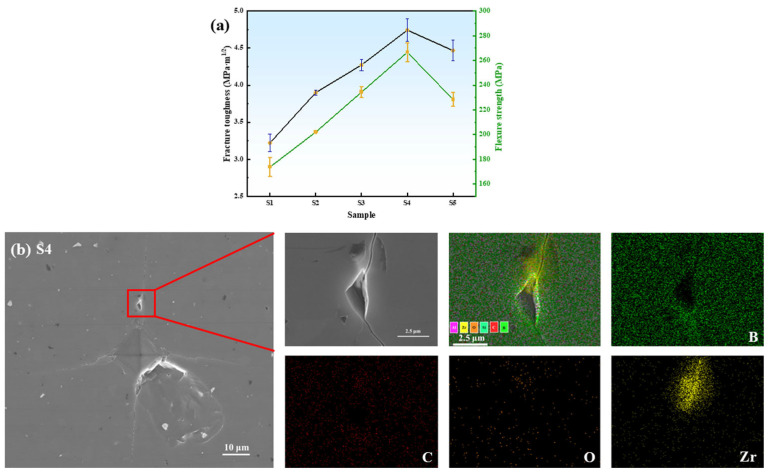
(**a**) Fracture toughness and flexural strength plots of B_4_C samples with different ZrO_2_ additions and (**b**) SEM/EDS mapping of cracked S4 samples.

The fracture toughness and flexural strength of B_4_C ceramics are summarized in Figure 12a. From Sample S1 to S4, the fracture toughness increases from 3.22 to 4.74 MPa·m^1/2^ (47.2% improvement), with Sample S2 showing a 9.6% enhancement over S1. Flexural strength similarly improves with higher HTMT-ZrO_2_ content, peaking at 266.62 MPa for Sample S4. However, Sample S5 exhibits reduced fracture toughness (4.32 MPa·m^1/2^) and flexural strength (228.43 MPa), even lower than that of S3 (234.40 MPa). This degradation is attributed to excessive in situ formation of micron-sized ZrB_2_, which acts as stress concentrators, significantly lowering fracture energy [25]. As evidenced by the dimple-crack morphology in Sample S4, cracks propagating to ZrB_2_/B_4_C interfaces are deflected along grain boundaries due to residual stress from thermal expansion mismatch between ZrB_2_ and B_4_C phases during cooling (as shown in Figure 12b). This prolongs the crack propagation path, requiring greater energy dissipation and thereby enhancing fracture toughness.

**Figure 13:**
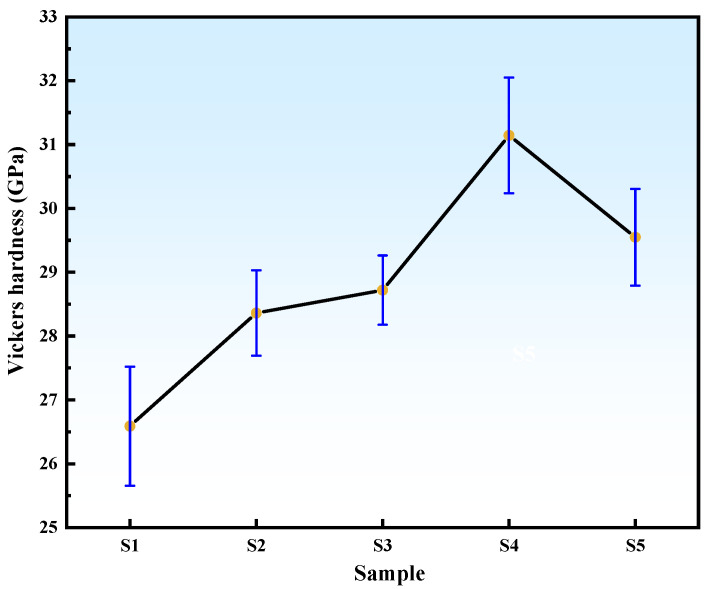
Vickers hardness of B_4_C samples with different ZrO_2_ additions.

The Vickers hardness of B_4_C samples with varying ZrO_2_ additions sintered at 2240 °C is shown in Figure 13. The reference Sample S1 exhibits a hardness of 26.59 GPa. Upon adding 2 wt% commercial and HTMT-synthesized nano-ZrO_2_, Samples S2 and S3 achieve enhanced hardness values of 28.36 GPa and 28.72 GPa, respectively. This improvement confirms that the secondary ZrB_2_ phase refines B_4_C grains, reduces porosity, and optimizes densification, while also introducing residual stress due to the difference in coefficients of thermal expansion between B_4_C and ZrB_2_, thereby enhancing hardness. Sample S4 attains the maximum hardness of 31.14 GPa, while Sample S5 shows a decline to 29.55 GPa, attributed to the increased proportion of low-hardness ZrB_2_ phase.

## 4. Conclusions

In this study, nano-sized ZrO_2_ powders were successfully synthesized via a novel high-temperature mechanochemical technology (HTMT) using ZrO(OH)_2_ as the zirconium precursor. ZrB_2_-B_4_C composites were subsequently fabricated by hot-pressing mixtures of B_4_C powder with varying contents of HTMT-derived and commercial nano-ZrO_2_. Key findings include:

(1) HTMT Process Optimization: The ZrO_2_ particle size exhibited a non-monotonic trend (initial decrease followed by increase) with rising ball milling temperature (400–700 °C) and prolonged duration (4–7 h). Optimal conditions were identified as 600 °C for 6 h, yielding ZrO_2_ powders with narrow size distribution (9.12 nm), low microstrain (10.64 × 10^−3^), high crystallinity, and excellent dispersion.
(2) Composite Performance: Compared to commercial nano-ZrO_2_, HTMT-ZrO_2_ demonstrated superior uniformity and dispersion, enabling enhanced densification of ZrB_2_-B_4_C composites. With increasing in situ-generated ZrB_2_ content, B_4_C crystallite size initially decreased (5.5 ± 0.3 μm at 2 wt%) and then increased (7.2 ± 0.4 μm at 6 wt%), while mechanical properties (fracture toughness: 4.74 MPa·m^1/2^; flexural strength: 266.61 MPa; Vickers hardness: 31.14 GPa) peaked at 4 wt% HTMT-ZrO_2_.
(3) This mechanism prolongs the crack extension path, resulting in a 47.2% increase in fracture toughness over B_4_C ceramics without ZrO_2_ addition.

The 4 wt% HTMT-ZrO_2_-B_4_C exhibited optimal comprehensive performance: density of 2.57 g·cm^−3^ (99.75% relative density), smooth microstructure, and balanced mechanical properties. This work establishes HTMT as a scalable, energy-efficient route for synthesizing high-performance ceramic composites, with implications for structural material design.

## Figures and Tables

**Table 1:** Sample composition.

Sample	Characteristics (wt%)			
	B_4_C	Commercially Available Nano-ZrO_2_	HTMT Nano-ZrO_2_	SiO_2_
S1	99	0	0	1
S2	97	2	0	1
S3	97	0	2	1
S4	95	0	4	1
S5	93	0	6	1

## Data Availability

The original contributions presented in this study are included in this article, further inquiries can be directed to the corresponding authors.
